# Screening and Initial Binding Assessment of Fumonisin B_1_ Aptamers

**DOI:** 10.3390/ijms11124864

**Published:** 2010-11-26

**Authors:** Maureen McKeague, Charlotte R. Bradley, Annalisa De Girolamo, Angelo Visconti, J. David Miller, Maria C. DeRosa

**Affiliations:** 1 Chemistry Department, Carleton University, 1125 Colonel By Drive, Ottawa, ON, Canada K1S 5B6; E-Mails: mmckeagu@connect.carleton.ca (M.M.); cbradle4@connect.carleton.ca (C.R.B.); david_miller@carleton.ca (J.D.M.); 2 Institute of Sciences of Food Production, National Research Council, via Amendola 122/O, 10 70126 Bari, Italy; E-Mails: annalisa.degirolamo@ispa.cnr.it (A.D.G.); angelo.visconti@ispa.cnr.it (A.V.)

**Keywords:** aptamer, fumonisin B_1_, mycotoxins, DNA, SELEX, toxins, maize, corn, binding affinity, molecular recognition

## Abstract

Fumonisins are mycotoxins produced by *Fusarium verticillioides* and *F. proliferatum*, fungi that are ubiquitous in corn (maize). Insect damage and some other environmental conditions result in the accumulation of fumonisins in corn-based products worldwide. Current methods of fumonisin detection rely on the use of immunoaffinity columns and high-performance liquid chromatography (HPLC). The use of aptamers offers a good alternative to the use of antibodies in fumonisin cleanup and detection due to lower costs and improved stability. Aptamers are single-stranded oligonucleotides that are selected using Systematic Evolution of Ligands by EXponential enrichment (SELEX) for their ability to bind to targets with high affinity and specificity. Sequences obtained after 18 rounds of SELEX were screened for their ability to bind to fumonisin B_1_. Six unique sequences were obtained, each showing improved binding to fumonisin B_1_ compared to controls. Sequence FB_1_ 39 binds to fumonisin with a dissociation constant of 100 ± 30 nM and shows potential for use in fumonisin biosensors and solid phase extraction columns.

## 1. Introduction

Molecular recognition plays an essential role in various biological and biomedical applications, particularly in biosensor development. In its simplest form, a biosensor consists of a specific molecular recognition probe targeting an analyte of interest and a means of converting that recognition event into a measurable signal. While antibodies have been the gold standard for molecular recognition elements for several decades, the relatively new technology of aptamers is emerging as a valuable molecular recognition tool. Aptamers are single stranded oligonucleotides that fold into distinct nanoscale shapes capable of binding to a target molecule. Aptamer-target recognition occurs through a combination of hydrogen bonding, electrostatic interactions, van der Waals forces and stacking interactions. As molecular recognition probes, aptamers have binding affinities and specificities that are comparable to, and in some cases even surpass those of monoclonal antibodies. The ability of aptamers to fold into distinct 3-D conformations allows high affinity binding and selectivity for their target. For example, dissociation constants in the nanomolar or picomolar range have been achieved with aptamers. Furthermore, aptamers can discriminate targets on the basis of subtle structural differences such as chirality [1] or the presence or absence of a single methyl group [2]. Targets for which aptamers have been developed range from small molecules, such as nucleotides [3], to complex macromolecules, such as proteins [4] and even whole cells [5]. In certain applications, aptamer technology offers several advantages over antibodies. First, high-purity aptamers can be chemically synthesized at a low cost and can be easily modified with dyes and labels without affecting their affinities. Second, aptamers are more chemically stable under most environmental conditions, have a longer shelf life, and can be reversibly denatured without loss of specificity. These properties make aptamers attractive in the development of low-cost, robust diagnostics and biosensors [6].

There is a growing need for rapid, inexpensive methods for the detection of contaminations in food and feed, both in the field and post-harvest. As molecular recognition is the cornerstone of sensing, there has been increased focus on the development of new molecular recognition probes for food-safety related targets. Toxic fungal metabolites, known as mycotoxins, can contaminate a wide range of agricultural commodities, and are high priority targets for the development of new molecular recognition probes and biosensors. It is estimated that at least 25% of the grain produced worldwide is contaminated with mycotoxins [7]. However, even small concentrations of mycotoxins can induce significant health problems including vomiting, kidney disease, liver disease, cancer and death. Thus, aptamers displaying high affinity and selectivity for such toxins would be useful in the development of sensitive biosensing systems enabling simple and rapid detection. The development of aptamers for mycotoxins is of particular interest as the antibodies used for mycotoxin detection are susceptible to denaturation in the presence of solvents commonly used in their extraction [8]. An aptamer for the mycotoxin ochratoxin A (OTA) has already been developed and integrated into several detection systems [9,10]. For example, the OTA aptamer has been used in the preparation of solid phase extraction columns for OTA clean-up from naturally contaminated wheat samples prior to liquid chromatographic (HPLC) analysis. Comparative analysis using traditional immunoaffinity clean-up procedures showed a good correlation between the two methods [11]. Additionally, the OTA aptamer has been incorporated into a colorimetric detection system utilizing gold nanoparticles, where accurate measurements can be done in less than 5 minutes [12]. Aptamer-based electrochemical and electrochemiluminescent sensors have also been developed using the OTA aptamer. These systems are sensitive, displaying detection limits of 30 pg/mL and 7 pg/mL, respectively [13,14]. Other mycotoxin aptamers are also in development worldwide [15,16].

The mycotoxin Fumonisin B_1_ (FB_1_) is a nephrotoxin in all species tested, a carcinogen in rodents and a reproductive toxicant in rodents and likely in humans [17]. The Joint Expert Committee on Food Additives and Contaminants of the WHO established a Provisional Maximum Daily Intake which is being applied in different areas as a function of corn-based food consumption and exposures [18]. Unlike food-contaminating bacteria, FB_1_ is not inactivated by cooking temperatures, therefore careful monitoring of cereals is essential prior to distribution for consumption [19]. Given its toxicity and prevalence, several analytical methods have been reported for the detection of FB_1_. These include enzyme-linked immunosorbent assay, capillary zone electrophoresis and high performance liquid chromatography technologies [20]. While these methods are effective, the need for highly trained personnel and expensive equipment make them inaccessible in all but the most well-equipped laboratories as well as impractical for rapid testing at country elevators, for example. Thus, a tight-binding aptamer for FB_1_ could be an important tool for the development of new fumonisin clean-up and detection systems.

**Figure 1.**
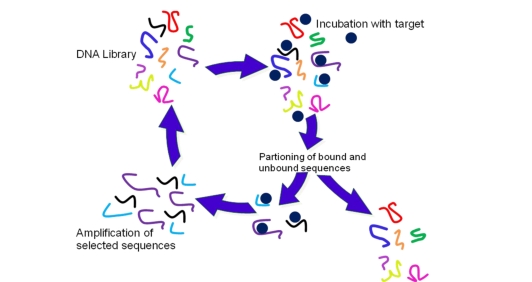
Schematic overview of the Systematic Evolution of Ligands by EXponential enrichment (SELEX) procedure.

Aptamers are discovered using an *in vitro* process called Systematic Evolution of Ligands by EXponential enrichment (SELEX), a procedure where target-binding oligonucleotides are selected from a random pool of sequences through iterative cycles of affinity separation and amplification (see Figure 1) [21,22]. The SELEX process begins with a large random oligonucleotide library (pool), whose complexity and diversity is dependent on the number of its random nucleotide positions [23].

During the SELEX procedure, separation of the binding DNA from the sequences lacking affinity is required. Typically, this is achieved by immobilizing the target of interest to a column matrix, usually agarose or sepharose, allowing easy partitioning of unwanted sequences through multiple washes. However, a more recent SELEX method termed FluMag SELEX makes use of magnetic beads as the solid matrix [24]. These beads can be surface-functionalized with almost any chemical group to allow for easy covalent attachment of a target. Additionally, only a relatively small amount of target is required for efficient conjugation to the beads compared to conventional column materials.

The next steps in SELEX include the elution of the binding sequences from the target and the polymerase chain reaction (PCR) amplification of those binders to yield an enriched pool for subsequent, more stringent, selection rounds. As the interactions that lead to molecular recognition between the binding sequences and the target are non-covalent in nature, relatively mild conditions can be used to separate the two species. Elution is typically performed using high concentrations of the target molecule, high temperatures or hydrogen-bond-disrupting compounds such as urea. Once separated from the target, the few binding DNA sequences are amplified by PCR to yield a practical amount of sample to continue the process

The enriched pool generated from this first round of selection is then subjected to further selection rounds that serve to increase the pool’s affinity for the target molecule (positive selections) or eliminate members of the pool that have affinity for undesirable compounds (negative selections). After several rounds, the enriched pool is cloned, sequenced, and characterized to find aptamers with the desired properties. Once these sequences are elucidated, solid-phase chemical synthesis is used to reproducibly synthesize aptamers in large quantities. During aptamer synthesis, a multitude of chemical modifications are possible, such as end-labeling for covalent attachment or backbone modification for increased stability, optimizing the aptamer’s properties for certain applications [25]. In this way, aptamers are ideal for many molecular recognition applications such as diagnostics and therapeutics. For example, aptamer-based biosensors have been developed for use in a variety of fields from medicine, to environmental monitoring and food safety [26,27].

The production of an aptamer for FB_1_ is the first step in the realization of inexpensive, robust and innovative detection technology that could offer a solution to the limitations of the existing detection techniques. In turn, this testing process may be simple enough to be performed by crop handlers and agricultural inspectors in the field, ultimately reducing the exposure of the mycotoxin to animals and humans around the globe. This paper discusses the selection of FB_1_-binding aptamer sequences and an initial assessment of their binding affinity.

## 2. Results and Discussion

### 2.1. Fumonisin B_1_ Immobilization

In order to easily separate binding DNA from non-binding DNA, immobilization of the target to a solid matrix is necessary. As magnetic separation technology is fast, simple and effective, the use of surface modified magnetic beads is a superior alternative to conventional immobilization on sepharose or agarose. In particular, substantially less target is required for efficient immobilization compared to the amounts required for column immobilization [28]. This is particularly important in the case of expensive or rare targets such as FB_1_. Carboxylic acid modified beads were chosen due to the simple coupling chemistry possible with the sole primary amine of FB_1_. Fumonisin B_2_, B_3_ and B_4_ all have this amine, which could allow for a similar aptamer selection protocol for these other members of the fumonisin family in the future. In addition, some evidence has shown that the primary amine is a critical part of the anti-FB_1_ antibody binding epitope. Conjugation via the amine would likely force our aptamer to exclude the amine in its binding epitope, which could allow for the development of antibody/aptamer sandwich assays [19].

Successful conjugation of the target FB_1_, was confirmed using HPLC. *O-*phthaldialdehyde (OPA) derivatized FB_1_ was fluorescently detected at a retention time of approximately six minutes. Quantification of unbound FB_1_ using a standard curve, measured as the total peak area, showed 1–5 nmol of target successfully conjugated to aliquots of 5 × 10^8^ beads. Comparison of the immobilized FB_1_ in the presence and absence of the required coupling agent, 1-ethyl-3-(3-dimethylaminopropyl) carbodiimide hydrochloride (EDC), showed that a small amount of FB_1_ (approximately 0.1 nmol) can non-specifically interact with the carboxylic acid surface activated beads.

### 2.2. Selection of Aptamers

Careful consideration was made when choosing the selection buffer for this experiment. Since the DNA backbone is polyanionic, the negative–negative charge repulsion may inhibit formation of complex structures in the absence of counterions such as Na^+^. This may ultimately impede binding with the target. Divalent cations in particular, such as Mg^2+^, block the negative charge repulsion and allow the backbone to form tighter folding. Interestingly, aptamers with higher affinity show less magnesium dependence than weaker binding aptamers [29]. Therefore, performing SELEX under low magnesium concentrations, such as physiological conditions (1–2.5 mM Mg^2+^), may increase the stringency of selection conditions, leading to the production of high-affinity binding aptamers. While the only other mycotoxin aptamer, the OTA aptamer, was selected under buffer conditions lacking calcium, an increase in binding affinity was observed upon the addition of calcium ions [9]. It is suggested that the OTA forms a coordination complex with divalent ions such as calcium with the aid of the carboxyl and hydroxyl groups. Calcium was included in the buffer for this experiment to allow for a similar coordination complex that may form between neighboring carboxyl groups in fumonisin. Many published aptamers fold into g-quadruplex structures such as the thrombin-binding aptamer [4], the DNA aptamer for hematoporphyrin [30] and the HIV-1 integrase aptamer [31]. Potassium was included in the selection buffer since monovalent cations, especially K^+^, are required to help stabilize g-rich sequences into these guanosine tetrads.

While aptamers have been selected using a variety of random region lengths within the initial library, a 60 nucleotide length was chosen for these experiments. A minimum of 25 nucleotides should be employed to achieve a diversity of 10^15^ different sequences. Generally, as the length of the random region increases, the structural complexity of the library increases. This increase in complexity allows for the presence of complex structures such as 4 and 5-way junctions, which are often high-affinity binders [32]. For this reason, lengths over 200 nucleotides have been used. However, practical limitations reduce the number of initial sequences that can actually be used for the first selection round to 10 nmol or less. Therefore, as the length of the random region increases, sequence space becomes substantially under-sampled. An initial library with 60 random nucleotides was chosen to strike a balance between these two competing factors.

When performing SELEX on a solid matrix for target immobilization, it is extremely important to ensure that any sequences displaying binding properties towards the matrix and/or the linker arm are eliminated. Once aptamers are generated, they are typically used to measure free target in solution, therefore any aptamer affinity derived from any partial binding to the matrix will reduce the functionality of the aptamer for sensing applications. For example, the published rhodamine aptamer displays a weaker binding to the target rhodamine when in solution compared to when it is immobilized on the matrix used in the selection [33]. To mitigate this, it is necessary to remove all sequences that display even weak binding or partial recognition to the matrix. Typical SELEX experiments do this by performing a pre-selection step prior to the first selection round to remove any initial sequences displaying this unwanted binding [34]. However, as mutation and evolution of the pool occurs with each selection and amplification step, we performed this negative selection prior to each round to ensure as much as possible that our pool was only binding to the target. To further enhance the selectivity of our aptamers, we performed our negative selection using various targets beginning at round 4. These negative selection steps, often called counter selections, remove any sequences from the pool that bind to structurally similar molecules, or molecules that may be found in a sample containing our target of interest. Jenison *et al.* found that using this counter selection technique generated an aptamer for theophylline that displayed a 10,000-fold weaker binding to caffeine, two molecules which differ only by a single methyl group [2]. Counter selections using l-homocysteine (rounds 5–10), l-cysteine and l-methionine (rounds 11–15) immobilized to the magnetic beads were used, since these amino acids could be easily coupled to the beads via the amino group. Pool sequences that only bind to any exposed carboxylic acid functional group would be removed by these negative selections. Similarly, in the last three rounds, l-glutamic acid (round 16–18) was then used as a counter selection to eliminate sequences with affinity for other similar carboxylic acid containing molecules. A negative selection using FB_2_ was not employed in our selections for several reasons. The difference between these two structures is very subtle: FB_2_ lacks the 11-hydroxy group. While aptamers have been selected that can recognize as small a difference as this, the counter selection against FB_2_ would likely severely reduce the DNA pool and eliminate many high affinity binders. The loss of high affinity binders in order to gain selectivity may not be worthwhile in this case as FB_2_ is often found in samples containing FB_1_. The European Commission maximum tolerable intakes include the total of FB_1_ and FB_2_ intake; therefore, total fumonisin is often detected [35]. Note that the absence of counter selections against FB_2_ does not guarantee that the selected aptamers will be able to bind both targets.

**Figure 2.**
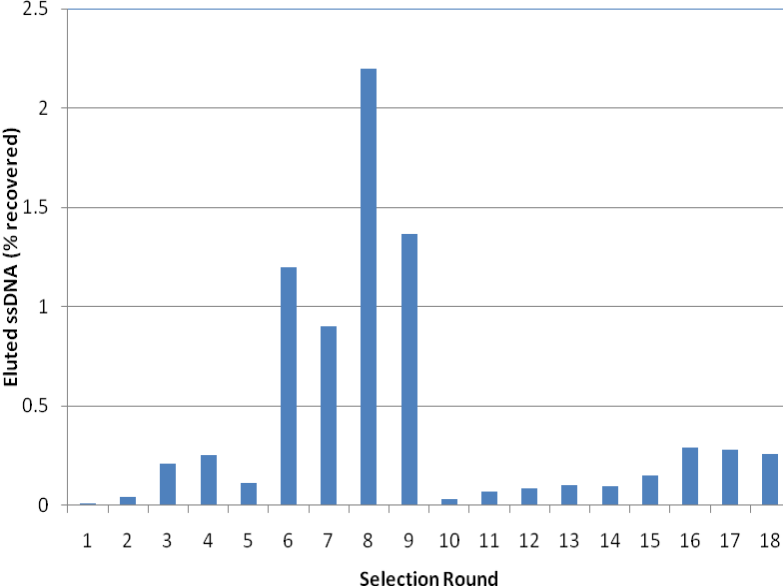
Percent recovery of binding ssDNA to the FB_1_ immobilized magnetic beads after each selection round. With any observable significant increase or plateau in percent recovery, increasingly stringent selection conditions were implemented the following round (see Section 3 and Table 1).

To assess the enrichment of the library for target binding, as well as to determine when increased stringency could be applied to the selection, the percent binding of the DNA pool was monitored after each round using fluorescence and absorbance measurements (see Figure 2). Typical selection matrices include μmol amounts of target [36]. However, due to the cost and availability of the target FB_1_, as well as the possibility of using minute concentrations of target with the magnetic beads, our selection matrix contained a maximum of 5 nmol of target. In the later rounds, the amount of target available for binding could be as low as 20 pmol (1:5 ratio of FB_1_:DNA). This accounts for the seemingly low percent recovery of binding sequences with each round.

In the initial few rounds, the maximum amount of target immobilized beads (5 × 10^8^ beads) was used for selections. This was to ensure complete capture of binding sequences. However, every 4–5 rounds, the number of magnetic beads used for positive selection, and therefore the amount of target available for binding, was reduced 2- to 5-fold (see Table 1). This decrease in the concentration of the target increased the stringency of the selection round, allowing for competition between sequences within the selection. Competition for the fewer binding sites should ultimately yield tighter-binding aptamers. Tok *et al.* described a novel method to generate DNA aptamers by using a single target-immobilized microbead. They found that the weak binding aptamers were displaced by the higher affinity aptamers. After only two selection cycles, aptamers displaying dissociation constants in the low nanomolar range were obtained [37].

Two other conditions were manipulated to increase the stringency of selections. First, non-binding aptamers were washed away with column buffer (prior to elution) only three times in the initial rounds. As the number of rounds increased, this was increased to eight washing steps to wash away not only the non-binding sequences but also any weaker-binding sequences. Second, the binding incubation time was slowly decreased as the selections progressed (see Table 1). In the initial rounds, a one hour period was used to allow for slow folding or refolding in the presence of the target. However, in the last rounds, 20 minute incubations were used since higher affinity binding species can be readily selected within a short equilibration time [34].

Several methods are possible for the elution of target-binding sequences. Direct cleavage of the target-bound aptamer from the bead was not possible in this case. While elution by affinity with free ligand is typically the method of choice [34], the amount required for successful elution for each round (often up to 50 mM) of 98% purity would cost over $5,000. Typically, the target used for elution should be as pure as possible: this would cost closer to $12,000 if obtained through a standard chemical supply house. For this reason, the elution methods available included changing the ionic strength or use of denaturants. We found that simply changing the ionic strength by the addition of metal chelators, such as (Ethylenedinitrilo)tetraacetic acid disodium salt (EDTA), or using deionized water, only allowed removal of weak binders. This is not surprising as some aptamers have been shown to maintain their binding in the absence of Na^+^ and K^+^ cations [9]. To fully remove the high affinity binders, several long elutions with 7 M urea at 90 °C were required. However, to ensure our final aptamers would bind to FB_1_ free in solution, elution with pure FB_1_ was performed in the last two selection rounds. As high affinity binders in these late rounds of SELEX should have very long residence times, a few long elution times were performed to completely remove all bound species. Following elution in these last two rounds, extensive clean-up to remove the FB_1_ from the binding sequences was required as the FB_1_ inhibited PCR and quenched fluorescence measurements.

Six unique sequences were obtained after cloning from round 18 (see Table 2). Surprisingly, sequences having the highest incidence (FB_1_ 31 and FB_1_ 39) have the lowest G content. Typically, aptamers are G rich, for example, the DNA aptamer for OTA (47% G) [9], the DNA aptamer for ATP (45%) [3], the DNA cocaine aptamer (32%) [38] and the DNA aptamer for fibronectin (33%) [39].

**Figure 3.**
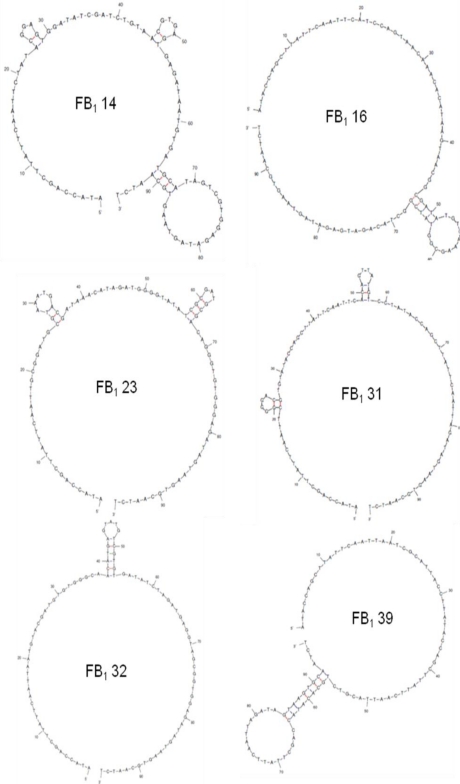
Secondary structure prediction for the full length clones. Image generated by Mfold.

The selected sequences were folded using Mfold [40] (see Figure 3). The resulting predicted secondary structures are all of very low complexity; all sequences are 1-way junction structures. While this is typical of several aptamers (almost a quarter of all aptamers are 1-way junctions [32]), some evidence has shown that higher complexity can lead to higher affinity [41]. For this reason, attempts have been made to increase the structural complexity of the initial libraries used in SELEX, which would result in more complex aptamers [32]. At this early stage, however, it is unclear if the large, initially unstructured regions of these sequences are involved in target binding, nor is it known whether these stretches fold in the presence of the target molecule as a result of new molecular interactions. These questions will be addressed in our future work.

### 2.3. Binding Assays

**Figure 4.**
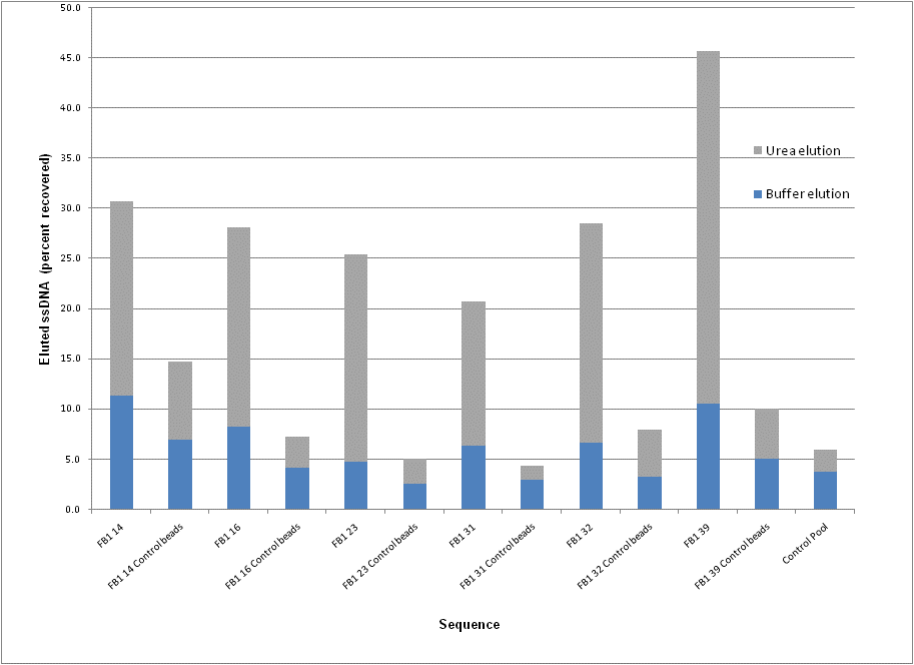
Binding assays of the individual aptamer sequences against FB_1_ beads compared to the unmodified beads (labeled as control beads). The percent of DNA eluted with buffer at 90 °C is in blue, the DNA eluted using 7 M urea at 90 °C is in grey. The unenriched (Control Pool) was tested on the FB_1_ modified beads as a comparison.

Each aptamer clone was tested for its ability to bind to FB_1_ derivatized beads using a fluorescence binding assay. This allowed for comparative determination of the relative binding ability of each aptamer generated in the selection experiment. The selection conditions used in the SELEX experiment were used for these binding assays, using aliquots of FB_1_ derivatized beads as the target. To remove any non-binding or weakly-bound DNA, many washes were performed. Bound DNA was eluted first with column buffer at 90 °C. Elution using high temperatures is sufficient to denature most target-DNA interactions; however, the addition of the denaturant urea, in our experience, is able to displace even the strongest, non-covalent, interaction. The comparison of binding DNA eluted only with 90 °C buffer compared to 90 °C urea can be found in Figure 4. The specific binding of the aptamers to the target was compared to binding of the unenriched initial library (Control Pool). Binding assays were also performed on unmodified (control) magnetic beads. This was done to ensure that the individual clones were binding specifically to FB_1_ and not simply non-specifically interacting with the surface of the magnetic beads.

Control pool, before any enrichment, has a small degree of non-specific affinity for the beads, as evidenced by the low percentage of DNA bound. This amount is comparable to the non-specific binding seen between each of the sequences and the unmodified beads (Control Beads). For most sequences, 50% or more of the DNA non-specifically bound to the unmodified beads can be removed using the 90 °C buffer, indicative that these interactions are relatively weak. The opposite is true in the case of the aptamer sequences and their affinity for FB_1_ beads. Across all sequences, a 2- to 5-times increase in affinity is seen in the presence of the target, and all required high concentrations of 90 °C urea in order to remove the majority of the bound sequences. The observed percent binding of the aptamer sequences is sizeable for this type of assay. A similar binding assay performed using streptavidin conjugated magnetic beads by Stoltenburg *et al.* showed a maximum percent binding of 21% for their aptamer clones. Despite their lower binding percents observed, their aptamers were found to bind to the target with dissociation constants in the low nanomolar range [24].

Interestingly, FB_1_ 39, which yielded the highest binding to the target, contains the lowest guanosine content (only 8%). As mentioned earlier, this is atypical of most aptamers. This sequence is slightly T enriched (35%) compared to C (27%) or A (30%). While less common, there is some precedent for T-rich aptamer sequences. For example, the DNA aptamer for tubulin is a T-rich aptamer displaying binding in the micromolar range. The random library used in their selection was also able to generate G-rich aptamers for other targets such as chitin, indicating that the T-rich aptamer property is specific for the target type and not simply a chance event due to the nature of the original pool [42].

**Figure 5.**
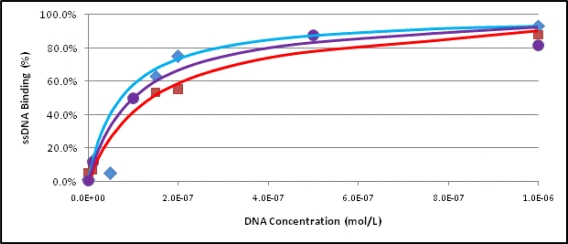
Binding curves for the aptamer sequence FB_1_ 39. Three trials were performed to obtain saturation curves used to determine the dissociation constant (*K*_D_). Bound fluorescein-labeled aptamer was measured by fluorescence (excitation at 490 nm, emission at 518 nm).

The binding affinity of sequence FB_1_ 39 was investigated in more detail and was found to interact with immobilized FB_1_ with a dissociation constant, *K*_D_, of 100 ± 30 nM (see Figure 5). Therefore, this full length aptamer clone displays a high affinity for this relatively small molecular target. Comparable binding of the aptamer to the target when in solution is expected and will be determined in our future work. Further analysis will also attempt to identify the minimal target-binding sequence to shorten the aptamer and improve binding while lowering the aptamer’s production cost.

As with the OTA aptamer, the effect of binding in an increased concentration of calcium will also be studied. The initial full length aptamer for OTA displayed a *K*_D_ of 360 nM. After identifying the minimal binding sequence and adding calcium to the binding buffer, the OTA aptamer displayed a *K*_D_ as low as 54 nM. A similar situation may be expected with our FB_1_ 39 sequence. Therefore, dissociation constants for the final minimal aptamer will be determined and cross-reactivity tests will be run against other mycotoxins such as fumonisin B_2_. Stability of the aptamer under typical fumonisin extraction conditions and the feasibility of incorporating the aptamer into solid phase extraction columns and biosensor systems will be examined.

## 3. Experimental Section

### 3.1. Reagents

DNA synthesis reagents were purchased from Glen Research (Sterling, VA, U.S.). Magnetic beads were purchased from Bangs Laboratories Inc. (Fishers, IL, U.S.). Fumonisin B_1_ was produced and purified in our laboratory according to Miller *et al*. [43]. All PCR and electrophoresis components were purchased from BioShop Canada Inc. (Burlington, ON, Canada). Chemicals for buffer solutions and all other purposes were purchased from Sigma-Aldrich (Oakville, ON, Canada).

### 3.2. Derivatization of Magnetic Beads

ProMag 3 Series Carboxylic acid Surfactant Free Bangs magnetic beads were covalently modified with fumonisin B_1_ through its primary amine according to the instructions from the beads manufacturer. Aliquots of 500 μL (approximately 5 × 10^8^ beads) were washed several times with Coupling Buffer (0.1 M K_2_HPO_4_, 0.15 M NaCl (pH 5.5)) and the beads were magnetically separated from the supernatant using a Dynal MPC-S, 6 × 1.5mL tube magnet. 500 μL of a 0.05 mM FB_1_ solution was reacted with the beads both in the presence and absence of excess 1-ethyl-3-(3-dimethylaminopropyl)carbodiimide (EDC) hydrochloride (∼5 mM) for 60 minutes. To determine successful immobilization, unbound FB_1_ remaining in the supernatant was measured using HPLC with fluorescence detection after derivatization with OPA reagent as described by Visconti *et al.* [44]. Separation was achieved with a C_18_ reverse phase HPLC column (5 μm, 250 × 4.6 mm) using a methanol: 0.1 M NaH_2_PO_4_ (77:23 v/v, pH 3.35) solvent system. Bound FB_1_ was calculated as the difference between the initial and unbound concentrations in the supernatants. Separate aliquots of beads were derivatized with one of l-homocysteine, l-cysteine, l-methionine or l-glutamic acid for counter selection steps.

### 3.3. DNA Library and Primer Synthesis

The starting DNA library was synthesized on a 1 μmol scale on a MerMade 6 Oligonucleotide synthesizer (BioAutomation Corporation, USA) using standard phosphoramidite chemistry. The sequence is 96 nucleotides in length, containing a central region of 60 random nucleotides flanked by two primer binding sites necessary for PCR and cloning: **5**′-**ATACCAGCTTATTCAATT**-N_60_-**AGATAGTAAGTGCAATCT-3**′. The following primers used for amplification and cloning of the selected oligonucleotides were synthesized: Primer1: 5′- ATACCAGCTTATTCAATT-3′ and Primer2: 5′-AGATTGCACTTACTATCT-3′. Modified primers were also synthesized to allow for fluorescent labeling of the binding DNA and separation of strands on a denaturing polyacrylamide gel: ModPrimer 1: 5′-fluorescein-ATACCAGCTTATTCAATT-3′ and ModPrimer 2 5′-poly-dA20-HEG-AGATTGCACTTACTATCT-3′. All synthesized DNA was purified through denaturing polyacrylamide gel electrophoresis (12%) followed by clean-up with Amicon YM-3 Centrifugal Filter Devices.

### 3.4. SELEX Experiments

Fresh aliquots of FB_1_ derivatized beads and unmodified beads or beads modified with a counter selection target were washed 5-times with 0.5 mL of Column Buffer (100 mM NaCl, 20 mM Tris, 2 mM MgCl_2_, 5 mM KCl, 1 mM CaCl_2_, pH 7.6) before each SELEX round. Prior to the first selection, the pool (2 nmol) was suspended in 0.5 mL of column buffer and heated for 10 minutes at 90 °C then cooled at 4 °C for 15 minutes, followed by incubation at room temperature for at least 7 minutes. In subsequent rounds, 100 pmol of pool was used. The pool was first incubated with either unmodified beads or beads derivatized with counter selection target molecules with mild shaking for 60 minutes. Only DNA lacking affinity for the unmodified beads or counter selection targets was collected by removal of the bead supernatant. This pool, devoid of sequences displaying non-specific affinity, was subjected to the same heating and cooling process described above, followed by incubation with a varying amount of FB_1_ derivatized beads and for a specific amount of time, depending on the stringency of binding selection desired (see Table 1). Following incubation, sequences with little to no affinity for FB_1_ were removed by washing 3–8 times with 0.1 mL of Column Buffer. DNA bound to the FB_1_ was then removed with 7 M urea at 90 °C. Five elution fractions of 0.1 mL each, were collected, purified using Amicon Ultra 3 KDa 0.5 mL centrifugal filters and quantified using UV-visible spectroscopy and/or fluorescence. In the final two selection rounds, binding DNA was removed with five 30 minute elutions using 0.2 mM FB_1_.

The entire selected oligonucleotide pool was amplified in 15–30 parallel PCR reactions for the first round, in subsequent rounds only 90% of the selected pool was amplified with the remaining 10% set aside for analysis and in case of PCR failure. Each reaction consisted of 0.1 M Tris HCl pH 9, 50 mM KCl, 1% Triton X-100, 2.0 mM MgCl_2_, 0.2 mM dNTP mix, 1 μM each modified primer and 5 units of Taq DNA polymerase. The DNA was initially melted for 10 minutes at 94 °C, followed by 25 cycles of 94 °C (1 min), 47 °C (1 min) and 72 °C (1 min). Final extension occurred at 72 °C for 10 minutes after the last cycle. In rounds 10–18, asymmetric PCR (1:50 ratio of primers, 35 cycles) was performed to facilitate the isolation of the aptamer strand from the complement. PCR products were dried down, heated at 55 °C for 5 minutes in the presence of formamide and run through a 12% denaturing PAGE to separate the double stranded product. The fluorescein labeled DNA strand (the selected sequences) could be identified using an Alpha Imager UV-illuminator. The corresponding DNA bands were cut from the gel and extracted using the freeze/rapid thaw method described by Chen and Ruffner [45] in 10 mM Tris HCl buffer, pH 7.4. The DNA was then purified using the Amicon centrifugal filters, quantified and re-suspended in column buffer and could be used in the next selection round.

Enrichment of the DNA pool was assessed throughout the rounds of selection by monitoring the percent of DNA pool binding to the FB_1_ derivatized beads using UV-vis and fluorescence measurements. After round 16, no additional enrichment was observed; therefore a total of 18 selection rounds were performed.

### 3.5. Cloning and Sequencing

The enriched DNA pool obtained from SELEX round 18 was PCR amplified using the unmodified primers (Primer 1 and 2) and cloned using the StrataClone PCR Cloning Kit (Agilent Technologies, Mississauga, ON, Canada). Positive colonies (white) were picked and a small portion of the plaque was removed carefully and transferred to the Templiphi sample buffer. DNA was prepared for sequencing by rolling-circle amplification using the TempliPhi Amplification kit (GE Healthcare, Canada). These samples were sent for full service sequencing at the University of Calgary University Core DNA Services (AB, Canada). The 6 unique sequences were tested for binding to FB_1_.

### 3.6. Binding Assays

The fluorescein-labeled (6-FAM) sequences to be tested (6 unique sequences obtained from the cloning experiment and some unenriched pool prior to SELEX) were synthesized on a 1 μmol scale using standard phosphoramidite chemistry. Sequence synthesis was verified through molecular weight verification using electrospray ionization (ESI) mass spectrometry (Novatia LLC, Monmouth Junction, NJ, USA). Each sequence was heated to 90 °C for 10 minutes, cooled to 4 °C and incubated at room temperature for 7 minutes. Aliquots of 2 × 10^8^ FB_1_ derivatized beads were washed with column buffer and incubated for 30 minutes at room temperature with 9 to 15 pmol of each DNA sequence separately. Non-binding DNA was removed with several washing steps using column buffer. Bound DNA was eluted first with two 0.1 mL fractions of column buffer at 90 °C for 10 minutes each. To ensure complete removal of bound DNA, elution using two fractions of 0.1 mL 7 M urea at 90 °C for 10 minutes was also performed. The percent of DNA eluted with column buffer and urea were determined using fluorescence (excitation at 490 nm, emission at 518 nm) and calculation using calibration curves. The same experiment was performed on unmodified beads to ensure the observed binding of sequences was specific to the target.

### 3.7. Determination of the Dissociation Constant

The dissociation constant was obtained for aptamer sequence FB_1_ 39. Binding assays were performed as above, by exposing varying concentrations of aptamer (1 nM to 1 μM) to 2 × 10^8^ FB_1_ derivatized beads in column buffer. Non-binding DNA was removed by washing five times. Bound DNA was eluted with two 0.1 mL fractions of column buffer at 90 °C for 10 minutes each. The fluorescence of the eluted aptamer was recorded and the dissociation constants were evaluated by minimizing the residual values between calculated and observed experimental Δfluorescence data using the solver feature of Microsoft Excel [46]. This experiment was performed three times to obtain the reported dissociation constant.

## 4. Conclusions

Eighteen rounds of increasingly stringent selection conditions were performed in order to obtain DNA sequences displaying high binding affinity to fumonisin B_1_. Of the six individual sequences obtained from the SELEX experiment, several candidates displayed superior binding to the target compared to controls. Aptamer sequence FB_1_ 39, displaying low complexity and an unexpectedly low percent of G, displayed the highest percent binding to the target. This sequence, displaying a dissociation constant in the nanomolar range, will be further processed to optimize the length and binding affinity to fumonisin. This aptamer is a promising tool for use in fumonisin clean-up and detection systems to efficiently detect low levels of fumonisin in food commodities. This aptamer may allow the development of a testing process simple enough to be performed by maize handlers and agricultural inspectors in the field and greatly reduce the exposure of animals and humans to FB_1_.

## Figures and Tables

**Table 1.** Summary of selection round conditions.

**Selection Round**	**Negative Selection Used**	**Number of FB_1_ derivatized beads used**	**Incubation time with FB_1_ derivatized beads(min)**
1	Unmodified	5 × 10^8^	60
2	Unmodified	5 × 10^8^	60
3	Unmodified	5 × 10^8^	60
4	Unmodified	5 × 10^8^	60
5	l-homocysteine	1 × 10^8^	60
6	l-homocysteine	1 × 10^8^	60
7	l-homocysteine	1 × 10^8^	45
8	l-homocysteine	1 × 10^8^	45
9	l-homocysteine	1 × 10^8^	45
10	l-homocysteine	5 × 10^7^	45
11	l-cysteine and l-methionine	5 × 10^7^	45
12	l-cysteine and l-methionine	5 × 10^7^	30
13	l-cysteine and l-methionine	5 × 10^7^	30
14	l-cysteine and l-methionine	5 × 10^7^	30
15	l-cysteine and l-methionine	1 × 10^7^	30
16	l-glutamic acid	1 × 10^7^	30
17	l-glutamic acid	1 × 10^7^	30
18	l-glutamic acid	1 × 10^7^	20

**Table 2.** Full length sequences obtained after selection round 18.

**Sequence**	**5**′**-ATACCAGCTTATTCAATT-N_60_-AGATAGTAAGTGCAATCT-3**′	**G Content (%)**	**Sequence Incidence (% of Total)**
**FB_1_ 14**	CTATACGGAGTGGATATCGATCTGTAACGT GAGTGAGATAATGTGATGCATAGTCGTGG	32	12.5
**FB_1_ 16**	CATCCAGTAACAAACACATAAGTAACGGC GATATGTCAAAGCGGTATCGGCTACAGATG	22	12.5
**FB_1_ 23**	GCGGATGCGTAAATGACGATAAACATAGAT GGGGTATATCGCGATGCGACAGGGTGT	35	12.5
**FB_1_ 31**	CGGGGACGTGTATACCAGCTTATTCAATTC ACAGTTATGTCCTATACCAGCTTATTCAATT	17	25
**FB_1_ 32**	AATGTACGATGTGTGGGCAACATGAGTATG TCGTGTGATATCTAGATGAGGTAGCGGTGG	37	12.5
**FB_1_ 39**	AATCGCATTACCTTATACCAGCTTATTCAAT TACGTCTGCACATACCAGCTTATTCAATT	8	25
